# Bilateral Gonadal Cysts and Late Diagnosis of Androgen Insensitivity Syndrome Treated by Laparoscopic Gonadectomy

**DOI:** 10.1155/2011/230845

**Published:** 2011-07-13

**Authors:** D. Tourlakis, J. Schnabel, N. Fersis

**Affiliations:** Department of Obstetrics and Gynecology, Klinikum Chemnitz gGmbH, Flemmingstraße 4, 09116 Chemnitz, Germany

## Abstract

*Background*. Complete androgen insensitivity syndrome is a rare syndrome in which the uterus is absent and testes rather than ovaries are present. Patients usually visit a gynecologist due to primary amenorrhea. *Case*. A forty-eight-year-old woman with lower abdominal pain and anamnesis of uterus agenesis was operated on due to bilateral cystic masses. A 5 × 3 × 1.2 cm left adnexal cyst revealed the presence of a serous cyst with a hypoplastic ductus deferens. A smaller cyst of the right adnexa revealed immature testis tissue with Leydig-cell hyperplasia. After karyotype and hormonal examinations, laparoscopic gonadectomy was performed. *Conclusion*. Attention should be paid in all cyst-removing operations in cases of uterus agenesis, due to the high incidence of malignancy. Not of less importance is the issue of informing the patient in the most appropriate way.

## 1. Introduction

Complete androgen insensitivity syndrome is a type of male pseudohermaphroditism in genotypically XY and phenotypically female patients, in which there is a defect that prevents normal androgen receptor function. Due to the presence of the anti-Müllerian hormone, the internal genital female organs are absent, and testes rather than ovaries are present [1]. Patients with the formery called testicular feminization syndrome present to the gynecologist usually due to adnexal masses or primary amenorrhea.

The incidence of neoplastic formation is 52%, half of which are malignant, and usually occurs after puberty [2]. The risk of developing malignancy increases with age, reaching 33% at the age of 50 [3]. In order to prevent malignant transformation, both gonads should be removed after puberty. During the last years laparoscopic gonadectomy has become the method of choice but in case of cysts it should be performed with caution in order to remove them intact and prevent eventual tumor cell dissemination.

We report a case of bilateral cyst removal in a 48-year-old woman, followed by gonadectomy, both performed laparoscopically.

## 2. Case Report

A 48-year-old woman, presented to us complaining of lower abdominal pain. At the age of 16 she had been visited by a gynecologist due to primary amenorrhea, and uterus agenesis was diagnosed by ultrasound. No further examinations were made at the time. The patient, married for 13 years, had normal female external genitalia, mature secondary sexual characteristics, Tanner stage II breast development with pale areolae, and scanty pubic and axillary hair. In the gynaecological examination, vulva and perineum appeared normal, but no cervix was visible and the proximal part of the vagina ended blindly. During bimanual examination, a 5 cm large cystic mass was palpated at the left adnexal region and a small right adnexa was palpated in the proximity of the internal inguinal canal opening. Ultrasound confirmed the absence of the uterus and revealed a 5 cm cystic mass on the left and another smaller cystic mass of 3 cm at the right adnexa. No signs of malignancy were observed.

Laparoscopy was performed to remove the cysts using a 3-port technique, and they were sent to the pathologist. Particular attention was paid to remove the cysts in the endobag to prevent eventual tumor cell dispersion. The histology revealed immature testicular tissue (inactive, as prepubertal) and Leydig-cell hyperplasia in the 5 cm large cyst, and a serous cyst with on it part of a small ductus deferens, with no apparent testicular structures, at the right adnexal specimen.

After that, tumor markers and hormonal evaluation, and also cytogenetic analysis, were performed. CA-125, CEA, a-FP, and *β*-HCG were all found within normal limits. Serum testosterone levels were 44.77 nmol/L, elevated and similar to normal male values, gonadotropins were found within normal values (FSH : 11.6 U/L, LH : 13.8 U/L), LH/FSH quotient 1.2, SHBG : 80.0 nmol/L and TSHi on serum 0.527 mE/L, also within normal values. Chromosomal analysis was made on lymphocyte cultures from peripheral blood and revealed XY karyotype.

**Figure 1:**
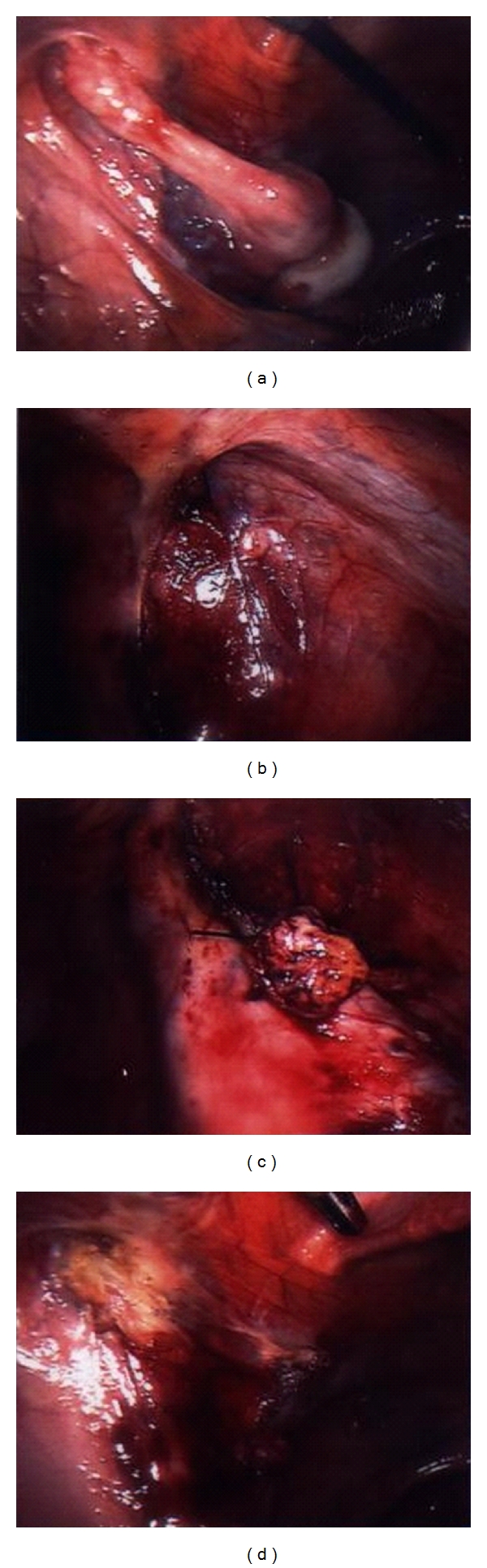
Laparoscopy pictures. (a) Left adnexa. (b) Right adnexa. (c) Left side after gonadal removal. (d) Right side after gonadal removal.

Intravenous urography excluded renal anomalies. The definitive diagnosis of androgen insensitivity syndrome was made and the patient was programmed for laparoscopic gonadectomy. The pedicles of the 2 adnexa, which were situated at the internal inguinal opening (Figures 1(a) and 1(b)), were first coagulated with bipolar diathermy, then cut with scissors, and finally ligated with a Roeder loop on each side (Figures 1(c) and 1(d)). As on the previous operation, due to the age of the patient and the statistically high malignant incidence, an endobag was used to remove the gonads after extending the right port. No complications occurred during the operation which lasted 30 minutes.

The histopathologic report revealed the presence of testicles on both sides, marked by Leydig-cell hyperplasia, and on the left side presence of small sites of ovarial stroma. 

Considering the age of the patient, followup was programmed and hormonal replacement therapy was given, and particular importance was attributed to the way of informing the patient. Due to the fact that she was married and evaluating the psychological effects that would provoke knowing that she is genotypically a man, we decided to inform the patient that she had some kind of gonadal dysgenesis and that the gonads would be removed for the high degeneration risks, deliberately omitting to inform her about the karyotype results.

## 3. Discussion

Androgen insensibility syndrome is the most common cause of male pseudohermaphroditism, responsible for 10% of the cases of primary amenorrhea [4]. Defects in the androgen receptor gene located on the X chromosome include absence of the gene that encodes for the androgen receptor and abnormalities in the binding domains of the receptor [1]. 

The major concern in these patients is the development of a neoplasia, most often a gonadoblastoma or a malignant dysgerminoma. Prophylactic gonadectomy is advised in the postpubertal period.

Laparoscopic gonadectomy offers the minimum psychological trauma which is of major importance in these women, for the rapid recovery and short hospital stay. It is a safe and simple procedure and is easily performed using bipolar coagulation forceps, Roeder loops and endobags, as long as the surgeon is cautious while removing the gonads or eventual cysts, to avoid tumor cell contamination, even more in patients of a certain age, as the one we present, where the malignancy incidence is much higher.

Hormonal replacement therapy was recommended, due to its protective effects [5]. In adolescent patients Slijper et al. advised step by step informing the patient of the diagnosis [6]. In cases as the present one, we advise to omit the karyotype analysis results, because the psychological impact would be enormous. 

We presented a rare case of bilateral cysts in a mature patient with complete androgen receptor insensitivity. Laparoscopic gonadectomy is effective and the importance of removing the cysts and later on the gonads intact is stressed.
